# Supplementary material for: Striatal D1 and D2 receptor availability are selectively associated with eye-blink rates after methylphenidate treatment
Source: Commun Biol. 2022 Sep 26;5:1015. doi: 10.1038/s42003-022-03979-5 (PMC9513088; doi:10.1038/s42003-022-03979-5)
Supplement: Supplementary file 2 — Supplementary Information [file 42003_2022_3979_MOESM2_ESM.pdf]

**Supplementary information for the manuscript**

# Striatal D1 and D2 receptor availability are selectively associated with eye-blink rates after methylphenidate treatment

Sukru B Demiral[1*], Peter Manza[1], Erin Biesecker[1], Corinde Wiers[2], Ehsan Shokri-Kojori[1], Katherine McPherson[1], Evan Dennis[1], Allison Johnson[1], Dardo Tomasi[1], Gene-Jack Wang[1], Nora D Volkow[3*]

1 National Institute on Alcohol Abuse and Alcoholism, Bethesda, MD, USA

2 Department of Psychiatry, University of Pennsylvania, PA, USA

3 National Institute on Drug Abuse, Bethesda, MD, USA

*Corresponding authors

***Supplementary Table 1.*** *Menstrual status of the female participants.*

| Nr | NNC | RAC_1 | RAC_2 | NNC last Menses | RAC1 last Menses | RAC2 last Menses | Contraception |
|---|---|---|---|---|---|---|---|
| 6 | 1/18/2018 | 1/18/2018 | 2/1/2018 | Post-Men | Post-Men | Post-Men | NA |
| 7 | 2/15/2018 | 2/15/2018 | 3/15/2018 | 2/11/2018 | 2/11/2018 | 3/12/2018 | NA |
| 11 | 5/3/2018 | 4/12/2018 | 5/3/2018 | Post-Men | Post-Men | Post-Men | NA |
| 12 | 4/17/2018 | 4/17/2018 | 4/26/2018 | 3/27/2018 | 3/27/2018 | 3/27/2018 | Unknown |
| 13 | 6/7/2018 | 5/10/2018 | 6/7/2018 | Post-Men | Post-Men | Post-Men | NA |
| 14 | 7/12/2018 | 7/12/2018 | 7/26/2018 | Post-Men | Post-Men | Post-Men | NA |
| 22 | 9/4/2019 | 3/7/2019 | 4/25/2019 | 8/25/2019 | 2/2/2019 | 3/30/2019 | NA |
| 27 | 5/9/2019 | 5/2/2019 | 5/9/2019 | 5/6/2019 | 4/10/2019 | 5/6/2019 | NA |
| 28 | 5/14/2019 | 5/14/2019 | 5/21/2019 | 4/26/2019 | 4/26/2019 | 4/26/2019 | Oral contraceptive |
| 43 | 12/10/2019 | 10/1/2019 | 10/8/2019 | 11/15/2019 | 9/27/2019 | 9/27/2019 | Oral contraceptive |
| 46 | 11/14/2019 | 11/14/2019 | 11/19/2019 | 10/24/2019 | 10/24/2019 | 10/24/2019 | NA |
| 47 | 1/14/2020 | 12/3/2019 | 1/14/2020 | 12/31/2019 | 11/29/2019 | 12/31/2019 | NA |

**Supplementary Table 2.** Head motion during PET Scans. Head motion was higher in the MP-D2R PET session than the PL-D2R PET session, t=2.10, p=0.044.

| | NNC | PL-D2R | MP-D2R |
|---|---|---|---|
| Framewise Displacement (mm) | 1.26(1.73) | 1.244(0.89) | 2.156(2.85) |

**Supplementary Table 3**. Head motion during eye tracking / resting state fMRI. The difference between MP day motion and PL day motion was not different (p>0.1) as found in paired 2-tailed t-test.

| | MP day resting fMRI | PL day resting fMRI |
|---|---|---|
| DVARS (%) | 0.083(0.042) | 0.072(0.024) |

**Supplementary Figure 1. Normality analysis of the MP and PL blink-rate distributions.** MP distribution was less normal than the PL distribution.

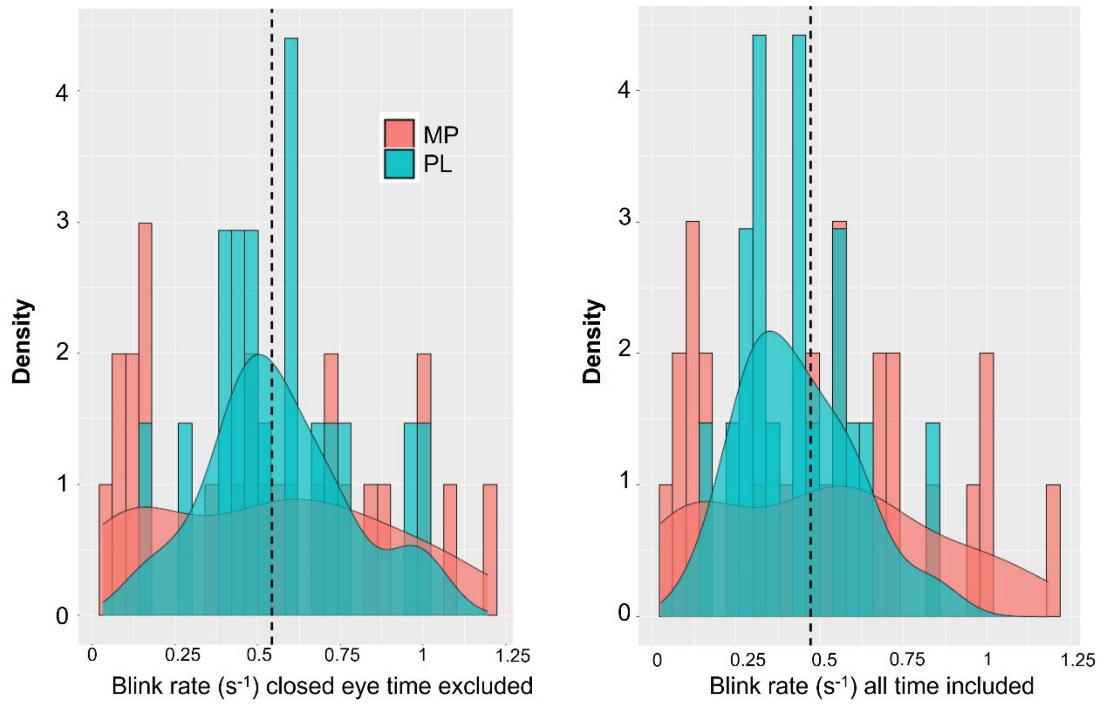


**Supplementary Figure 2. Kurtosis analysis.** When we compared the kurtosis of the two distributions against the permutation sample (10,000 permutations of PL and MP group blink rates), the actual kurtosis difference between the groups did not reach significance.

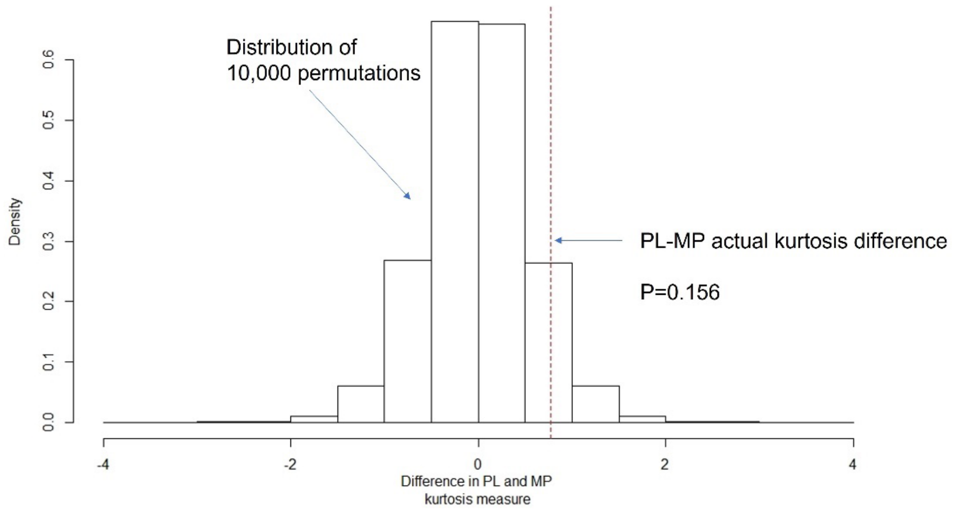

**Supplementary Figure 3. EBR difference analysis by taking all the recording times.**

This analysis did not reveal any effect of MP.

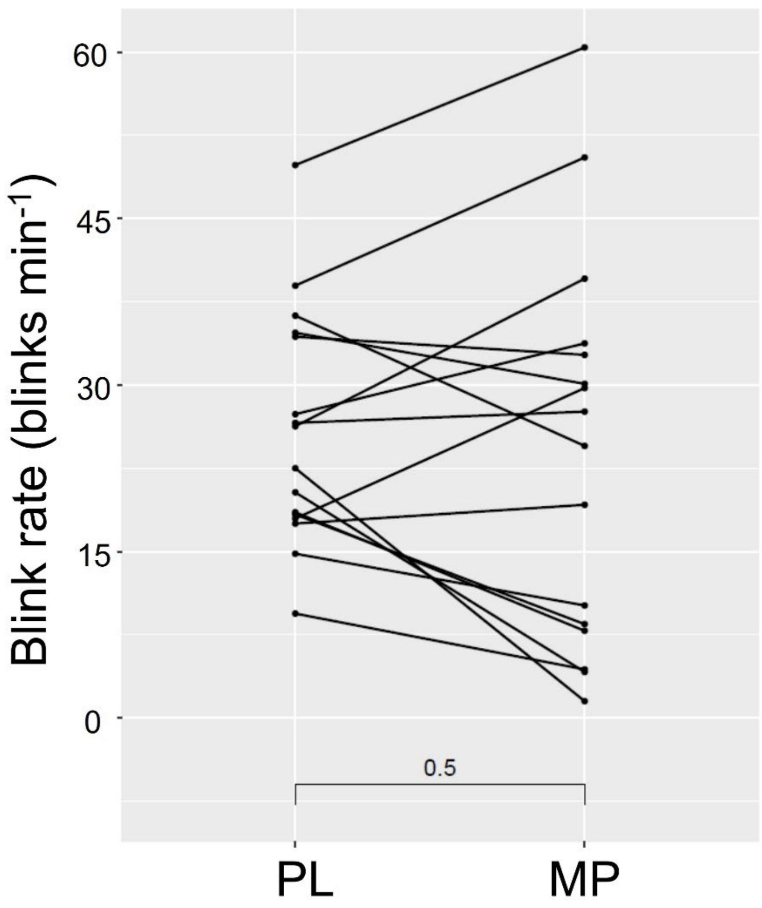


**Supplementary Note 1.** Blink rate distribution analysis.

We conducted Shapiro-Wilk test on each of the MP and PL sessions for the eye blink ra te measures collected with and without the eyes closed sections. The distributions did n ot violate normality:

MP blink rate; all times included
W = 0.93986, p-value = 0.147

MP blink rate; eyes closed time excluded
W = 0.93719, p-value = 0.1275

PL blink rate; all time included
W = 0.95876, p-value = 0.6082

PL blink rate; eyes closed time excluded

W = 0.97528, p-value = 0.9024

**Supplementary Note 2.** Model assumptions and additional models.

Multiple regression models assume the following: i) A linear relationship between the dependent and independent variables, ii) The independent variables are not highly correlated with each other, iii) The variance of the residuals is constant, iv) Independence of observation, v) Multivariate normality.

We conducted multiple regression models including eye closure rates (as well as age, gender and Raclopride/NNC) as explanatory variable(s) and blink rates (PL-BLR and MP-BLR) as response variables, and the results were similar to the models we reported in the manuscript. For instance a model with closure rate during the MP session, age, gender and MP-D2R as explanatory variables and MP-BLR as response variable was significant in putamen, $F(4,17)=9.68$, $R^2=0.695$, $p<0.001$; with model MP-EBR=-1.510+0.837*(MP-D2R)-0.305*(GenderMale)-0.005*(Age)+0.019(MPCLOSURE), revealed effects of MP-D2R, $\beta=0.837$, $p<0.001$ and gender $\beta=-0.305$, $p<0.01$, Males<Females), and no significant models for caudate and VS. A model with closure rate during the MP session, age, gender and PL-D2R as explanatory variables and MP-BLR as response variable was significant in caudate; $F(4,17)=3.25$, $R^2=0.298$, $p<0.05$; with model MP-EBR=-0.564+0.464*(PL-D2R)-0.303*(GenderMale)-0.001*(Age)+0.033(MPCLOSURE) with significant effects of PL-D2R, $\beta=0.464$, $p<0.05$ and gender $\beta=-0.303$, $p<0.05$, Males<Females), and no significant model fit for putamen and VS. In addition, multiple regression model including BL-D1R, gender, age and closure rate during MP session as explanatory variables and MP-BL as response variable was marginal for caudate ($F(4,17)=2.896$, $R^2=0.305$, $p=0.053$; with model MP-EBR=-0.538+0.499*(BL-D1R)-0.261*(GenderMale)+0.0004*(Age)+ 0.028(MPCLOSURE), where effect of BL-D1R was significant ($\beta=0.499$, $p<0.05$).

**Supplementary Note 3.** Regression model with interaction term.

We conducted a linear model with an interaction between Raclopride scans (COND: PL/MP) and BPnd (RAC) as explanatory variables and blink rate (BLR) as response variable. Significant interaction emerged only for putamen and not for caudate or VS. Here we present a summary of the results below. (We also provide this information in the supplementary material).

**Putamen:**

$F(3,38)=8.258$, $R^2=0.395$, $p<0.001$

Model: EBR=-1.612+0.747*(RAC)+2.655*(CONDpl)-0.896*(RAC*CONDpl)

Effects of RAC, $\beta=0.747$, $p<0.001$, CON, $\beta=2.655$, $p<0.01$, and RAC*COND, $\beta=-0.896$, $p<0.001$.

**Caudate:**

$F(3,38)=0.972$, $R^2=0.071$, $p=0.41$

Model: EBR=-0.248+0.312*(RAC)+0.623*(CONDpl)-0.241*(RAC*CONDpl)

**VS:**

$F(3,38)=0.132$, $R^2=0.016$, $p=0.93$

Model: EBR=0.417+0.050*(RAC)+0.388*(CONDpl)-0.149*(RAC*CONDpl)

**Supplementary Note 4.** Residual NNC activity on RAC scans.

Over 32 participants attending to the study, only 3 of them had NNC scan in a different day. Overall, 18 participants had their NNC scan on the same day as their MP RAC scan, and 11 participants had their NNC on the same day as their PL RAC scan. When we looked at the 25 subjects whose eye tracking data was available, we saw that 3 participants had NNC in a different day, 8 in PL RAC day, and 14 in MP RAC day.

**Supplementary Note 5.** PET Harmonization.

Our ComBat harmonization procedure assumed that the error terms may follow different normal distributions for the HRRT and PET-CT cameras, and that additive and multiplicative effects are not completely independent across ROIs/voxels but, rather, they share a common distribution. Such considerations prevent the use of standard linear models. Furthermore, ComBat uses an empirical Bayes framework to estimate the distribution of the effects for each camera. Missing values were handled first by automatically imputing missing data to find harmonization parameters with minimal errors.

Let the data come from 2 imaging cameras ($i$=1, 2), each containing $n_i$ scans, for voxels v=1, 2,…,p, leading to $n=\sum_{i=1}^{m} n_i$ total number of scans.

Let $y_{ijv}$ represent the PET measure of interest (i.e., DVR) in voxel $v$ for scan $j$ for camera $i$. ComBat posits location and scale (L/S) adjustment model as follows:

1) $y_{ijv} = \alpha_v + \mathbf{X}_{ij}\boldsymbol{\beta}_v + \gamma_{iv} + \delta_{iv}\varepsilon_{ijv}$

where $\alpha_v$ is the overall PET measure for voxel $v$, **X** is the n x K design matrix for the K covariates of interest (e.g. drug, age, gender). $\boldsymbol{\beta}_v$ is the voxel-specific vector of regression coefficients corresponding to **X.** The parameter-vector $\boldsymbol{\beta}_v$ was estimated using ordinary least squares (OLS). The terms $\gamma_{iv}$ and $\delta_{iv}$ represent the additive and multiplicative site effects of camera $i$ for voxel $v$, respectively. It is assumed that the error term $\varepsilon_{ijv}$ have mean zero and variance of $\sigma^2_v$. ComBat uses EB framework to improve the variance of the parameter estimates $\gamma_{iv}$ and $\delta_{iv}$. It estimates an empirical statistical distribution for each of those parameters by assuming that all voxels share the same common distribution. In that sense, information from all voxels is used to inform the statistical properties of the camera effects. More specifically, the camera-effect parameters are assumed to have the parametric prior distributions:

$\gamma_{iv} \sim N(\gamma_i, \tau^2_i)$ , and

$\delta^2_{iv} \sim$ Inverse Gamma $(\lambda_i, \theta_i)$

The hyperparameters $\gamma_i$ , $\tau_i^2$ , $\lambda_i$ ,$\theta_i$ are estimated empirically from the data as described in [24]. The final ComBat harmonization of PET measures then defined as:

2) $y_{ijv}^{ComBat} = ((y_{ijv} - \hat{\alpha}_v - \mathbf{X}_{ij}\hat{\boldsymbol{\beta}}_v - \gamma^*_{iv})/\delta^*_{iv}) + \hat{\alpha}_v + \mathbf{X}_{ij}\hat{\boldsymbol{\beta}}_v$

We conducted combat separately for each tracer for the PET measure of interest (e.g., BPnd) to harmonize the data across cameras. For [$^{11}$C]raclopride measures, since we had PL and MP treatments, we used drug (PL/MP), age and gender (M/F) as covariates in the model. For [$^{11}$C]NNC112 measures, we used age and gender as covariates.
