# Supplementary material for: Striatal D1 and D2 receptor availability are selectively associated with eye-blink rates after methylphenidate treatment
Source: Commun Biol. 2022 Sep 26;5:1015. doi: 10.1038/s42003-022-03979-5 (PMC9513088; doi:10.1038/s42003-022-03979-5)
Supplement: Supplementary file 3 — Reporting Summary [file 42003_2022_3979_MOESM3_ESM.pdf]

nature portfolio

Corresponding author(s): Sukru B Demiral & Nora D Volkow

Last updated by author(s): Sep 1, 2022

# Reporting Summary

Nature Portfolio wishes to improve the reproducibility of the work that we publish. This form provides structure for consistency and transparency in reporting. For further information on Nature Portfolio policies, see our Editorial Policies and the Editorial Policy Checklist.

## Statistics

For all statistical analyses, confirm that the following items are present in the figure legend, table legend, main text, or Methods section.

| n/a | Confirmed | |
|---|---|---|
| ☐ | ☒ | The exact sample size ($n$) for each experimental group/condition, given as a discrete number and unit of measurement |
| ☐ | ☒ | A statement on whether measurements were taken from distinct samples or whether the same sample was measured repeatedly |
| ☐ | ☒ | The statistical test(s) used AND whether they are one- or two-sided<br>*Only common tests should be described solely by name; describe more complex techniques in the Methods section.* |
| ☐ | ☒ | A description of all covariates tested |
| ☐ | ☒ | A description of any assumptions or corrections, such as tests of normality and adjustment for multiple comparisons |
| ☐ | ☒ | A full description of the statistical parameters including central tendency (e.g. means) or other basic estimates (e.g. regression coefficient) AND variation (e.g. standard deviation) or associated estimates of uncertainty (e.g. confidence intervals) |
| ☐ | ☒ | For null hypothesis testing, the test statistic (e.g. $F$, $t$, $r$) with confidence intervals, effect sizes, degrees of freedom and $P$ value noted<br>*Give P values as exact values whenever suitable.* |
| ☒ | ☐ | For Bayesian analysis, information on the choice of priors and Markov chain Monte Carlo settings |
| ☒ | ☐ | For hierarchical and complex designs, identification of the appropriate level for tests and full reporting of outcomes |
| ☐ | ☒ | Estimates of effect sizes (e.g. Cohen's $d$, Pearson's $r$), indicating how they were calculated |

*Our web collection on statistics for biologists contains articles on many of the points above.*

## Software and code

Policy information about availability of computer code

| | |
|---|---|
| Data collection | [11C]raclopride scans were performed on one of two scanners: a high-resolution research tomography (HRRT) scanner (Siemens AG; Germany) via MOLAR v2.2 software or Siemens Biograph mCT (Siemens Medical Solutions, Knoxville, TN, USA)<br>S/W version: VG62B. Eye blinks were measured while participants were undergoing resting MRI scans using an ASL (Applied Science Laboratories, previously known as Argus Science Inc., Bedford, MA) long-range LRO eye-tracker camera. High-resolution MRI scans were acquired on a 3.0T Magnetom Prisma scanner (Siemens Medical Solutions USA, Inc., Malvern, PA) with a 32-channel head coil. |
| Data analysis | Eye tacking blink detection was conducted using ET7 software system (Applied Science Laboratories, previously known as Argus Science Inc., Bedford, MA). Blink statistical analyses were conducted and plotted with R version 3.6.1. PET blink parametric analysis was conducted in SPM 12. |

For manuscripts utilizing custom algorithms or software that are central to the research but not yet described in published literature, software must be made available to editors and reviewers. We strongly encourage code deposition in a community repository (e.g. GitHub). See the Nature Portfolio guidelines for submitting code & software for further information.

# Data

Policy information about availability of data

All manuscripts must include a data availability statement. This statement should provide the following information, where applicable:

- Accession codes, unique identifiers, or web links for publicly available datasets
- A description of any restrictions on data availability
- For clinical datasets or third party data, please ensure that the statement adheres to our policy

Datasets collected and scripts used for the current study are available from the first or corresponding authors on reasonable request. Additional data processing scripts will be available at https://github.com/demiralsb/Blink-Arousal.

# Field-specific reporting

Please select the one below that is the best fit for your research. If you are not sure, read the appropriate sections before making your selection.

☐ Life sciences ☒ Behavioural & social sciences ☐ Ecological, evolutionary & environmental sciences

For a reference copy of the document with all sections, see nature.com/documents/nr-reporting-summary-flat.pdf

# Behavioural & social sciences study design

All studies must disclose on these points even when the disclosure is negative.

| | |
|---|---|
| Study description | Quantitative experimental |
| Research sample | Healthy population around Bethesda, MD region of USA. Age limit to participate was between 18-80. 20 males (mean age=40.8) and 12 females (mean age=44.9) attended to the study. |
| Sampling strategy | Random sampling. Sample size estimation showed a need for a sample of minimum of 15 subjects to yield power of 0.8, alpha=0.05, and expected mean eye-blink rate difference between placebo and stimulant conditions as 8 blinks/min (sd=10blinks/min; effect size 0.8) estimated for two tailed paired t-test. |
| Data collection | PET data was collected in the PET scanners while clinical personnel was monitoring participant for safety. Eye tracking data was collected while participant was in the MRI scanner lying supine position with a long-range infrared camera. Subject monitoring was handled from the MRI control room whileadditional safety cameras were placed in the scanner room. |
| Timing | From August, 2019 to August, 2021 |
| Data exclusions | participants with insufficient eye-tracking pupil detection or bad calibration performances were eliminated from data analysis. |
| Non-participation | No participants dropped participation. |
| Randomization | This is a repeated-measure design. Each subject received placebo as well as stimulant condition. |

# Reporting for specific materials, systems and methods

We require information from authors about some types of materials, experimental systems and methods used in many studies. Here, indicate whether each material, system or method listed is relevant to your study. If you are not sure if a list item applies to your research, read the appropriate section before selecting a response.

## Materials & experimental systems

| n/a | Involved in the study | |
|---|---|---|
| ☒ | ☐ | Antibodies |
| ☒ | ☐ | Eukaryotic cell lines |
| ☒ | ☐ | Palaeontology and archaeology |
| ☒ | ☐ | Animals and other organisms |
| ☐ | ☒ | Human research participants |
| ☐ | ☒ | Clinical data |
| ☒ | ☐ | Dual use research of concern |

## Methods

| n/a | Involved in the study | |
|---|---|---|
| ☒ | ☐ | ChIP-seq |
| ☒ | ☐ | Flow cytometry |
| ☒ | ☐ | MRI-based neuroimaging |

## Human research participants

Policy information about studies involving human research participants

| | |
|---|---|
| Population characteristics | See above. |
| Recruitment | Participants were recruited after initial screening via phone interview. |
| Ethics oversight | National Institute on Alcohol Abuse and Alcoholism, National Institutes of Health |

Note that full information on the approval of the study protocol must also be provided in the manuscript.

## Clinical data

Policy information about clinical studies

All manuscripts should comply with the ICMJE guidelines for publication of clinical research and a completed CONSORT checklist must be included with all submissions.

| | |
|---|---|
| Clinical trial registration | NCT03190954 |
| Study protocol | https://clinicalstudies.info.nih.gov/protocoldetails.aspx?id=17-AA-0114&&query= |
| Data collection | Data was collected at NIH, Bethesda campus, and in NIH facilities. |
| Outcomes | Primary outcome measure was spontaneous eye-blink rate and its association with PET images. |
